# Optimizing nursery conditions of the commercial kelp *Alaria esculenta*

**DOI:** 10.1007/s10811-025-03665-z

**Published:** 2025-09-27

**Authors:** Reina J. Veenhof, Rob Grisenthwaite, Alison Mair, Elaine Mitchell, Michele S. Stanley, Puja Kumari

**Affiliations:** 1 University of the Highlands and Islands, UHI House, Old Perth Road, Inverness, IV2 3JH UK; 2 Scottish Association for Marine Science, Oban, Argyll PA37 1QA UK; 3 SAMS Enterprise, Oban, Argyll PA37 1QA UK

**Keywords:** Phaeophyceae, Bulk cultivation, Gametogenesis, Photosynthesis, Reproduction, Seaweed aquaculture, Seeding

## Abstract

A global rise in kelp aquaculture has resulted in an increased demand for high quality and quantity kelp seedstock, yet many bottlenecks remain to sustainable scale-up solutions. One such bottleneck is increasing biomass quickly and efficiently in the nursery phase of the kelp cultivation cycle. The microscopic gametophyte is leveraged during this phase, using red light to stimulate vegetative growth and delay onset of gametophyte fertility until deployment. However, red light may make the gametophyte less photosynthetically efficient and thus slow growth. A solution may be growing gametophytes in blue light, which can increase production, while changing the nutrient composition of the media to repress fertility. Here we test the efficacy of light source (blue and red), two common media types (PES and f/2) and iron supplementation on the growth, fertility and photosynthetic parameters of *Alaria esculenta* gametophytes, a commercial kelp species. We found the highest biomass was achieved under blue light with iron-free f/2. Similarly, pigment concentration was higher in blue light and iron-free f/2, while gametophytes were found to have more shade-adapted pigment characteristics under red light. Red light strongly supressed formation of sporophytes and while withholding iron did not supress fertility in blue light, it did result in less malformed sporophytes. Based on this research, we recommend growing *A. esculenta* gametophytes in iron-free f/2. Failure of iron-free media to supress fertility in blue light may be due to iron retention of gametophytes or trace levels of iron in seawater, which warrants further research.

**Supplementary Information:**

The online version contains supplementary material available at 10.1007/s10811-025-03665-z.

## Introduction

There is increased commercial interest in the expansion of kelp aquaculture (Buschmann et al. 2017; Naylor et al. 2021). In the North Atlantic specifically, kelp cultivation has received considerable attention as a sustainable source of biomass for various downstream industrial applications such as food, feed, biostimulants, biofuels and pharmaceuticals (Kerrison et al. 2015; Veenhof et al. 2024; Yong et al. 2024). However, roadblocks exist that prevent further expansion of kelp aquaculture, mainly through lack of economic feasibility and scalability issues (Hafting et al. 2015; Hasselström et al. 2020). One particular roadblock pertains to controlling growth and fertility during the nursery phase of the kelp cultivation cycle (Coleman et al. 2022).

Kelps have a biphasic diplohaplontic life cycle alternating between microscopic haploid gametophytes and macroscopic diploid sporophytes. This is leveraged during the cultivation cycle, where the microscopic gametophytes are cultured in lab-based nurseries, while the adult diploid sporophytes are deployed on seeded lines in coastal waters (Kim et al. 2017). Both phases require optimization and a management framework for maximising production capacity (Ratcliff et al. 2017). The nursery phase of kelp cultivation is critical for successful deployment and consists of collecting fertile materials, releasing spores and bulking the gametophyte biomass before seeding lines or seeding spores directly on lines (Yarish et al. 2017). In order to meet increasing demand for seeded lines, methods for efficiently maintaining and upscaling gametophyte stock cultures in the nursery phase are essential (Coleman et al. 2022). Using gametophyte bulk culture has gained significant interest as it allows control over fertility and timing of deployment, creating some independence from the natural reproductive cycle (Ratcliff et al. 2017). During the bulking phase, vegetative growth of gametophytes can be rapid and is traditionally stimulated under red light and with added nutrient supply (Ebbing et al. 2022).

The quality of light in particular has long been recognised as a mechanism to control fertility in kelp gametophytes. Red light suppresses fertility while blue light stimulates fertility by promoting oogonia formation in gametophytes (Lüning and Dring 1975; Lüning and Neushul 1978). Changes in light however affect productivity of gametophytes, through changing the efficiency and composition of the photosynthetic apparatus (Veenhof et al. 2022). Kelps are adapted to live in a wide range of environmental conditions, including changing light intensity and wavelength with depth and season, so biochemical composition and functioning of the photosynthetic apparatus can change rapidly within the individual (Gerard 1988). This phenotypic plasticity allows kelps to adjust to attenuating light conditions by increasing or decreasing the number of pigments and thus photosynthetic efficiency (Henley and Dunton 1995). This reaction is often species-, life stage- and environment-specific (Gerard 1988, 1990; Altamirano et al. 2004; Blain et al. 2020; Wright and Foggo 2021). Generally, increased pigmentation and photosynthetic capacity is found in more shade-adapted kelps and the juvenile life stages, as an adaptation to living in light limited conditions under the shade of adult canopy and/or deep and turbid water (Gerard 1988; Altamirano et al. 2003).

Optimizing photosynthetic capacity and thus growth must be balanced with maintaining the gametophytes in their vegetative state during bulking in commercial nurseries. Gametophytes with delayed sexual reproduction can be maintained vegetatively under controlled conditions for long periods of time and provide additional possibilities for upscaling and breeding desired kelp traits for sustainable farming (Barrento et al. 2016; Ebbing et al. 2025). However, while red light is very effective in supressing fertility, it also slows growth as compared to blue or white light (Cuijuan et al. 2005; Xu et al. 2005). One way to achieve increased growth while still suppressing fertility may be by altering nutrient composition of the culture media. Removing iron from growth media can supress oogonia formation in kelp gametophytes (Motomura and Sakai 1984; Lewis et al. 2013; Iwai et al. 2015). Withholding iron under blue light may accelerate the gametophyte growth while also suppressing fertility during the bulking phase. The effect of iron on egg release is however, species-specific (Lewis et al. 2013) and has not been tested on some of the favoured species in Atlantic kelp aquaculture, such as *Alaria esculenta* to the best of our knowledge.

*Alaria esculenta* is a common temperate kelp that grows abundantly along the UK coastline and is currently a favoured species in aquaculture (Kraan 2020; Veenhof et al. 2024). Downstream application of *A. esculenta* include uses in feed- and food-industry, fertilizers as well as the extraction of bioactive, high-value compounds for pharma- and nutraceuticals (Blanco et al. 2023; Arlov et al. 2024). It is known to grow well in cold and clear waters and often favours more wave-exposed sites (Kraan 2020). Attenuating light conditions can alter the morphology of *A. esculenta* sporophytes (Ronowicz et al. 2022) and gametophytes can recruit and grow in higher intensity light conditions than more shade-adapted species (Müller et al. 2012; Silva et al. 2022). To meet the growing demand for *A. esculenta* seedstock, in this study, we trialled nursery cultivation methods to bulk *A. esculenta* gametophytes aiming to maximize growth while suppressing fertility. Specifically, we tested the combined effects of light, nutrient media and iron on the growth, fertility and photosynthetic capacity of *A. esculenta* gametophytes.

## Materials & Methods

### Culture collection and establishment of gametophyte cultures

*Alaria esculenta* gametophyte cultures were obtained from the seaweed nursery at the Scottish Association for Marine Science (SAMS). To establish these cultures, fertile sori tissues of *A. esculenta* were collected along the coast of Ellenabeich, Seil, Scotland (56°17'46.6"N 5°39'15.2"W) in August 2021. Fertile sporophylls were taken from the base of the blade, scraped clean of epiphytes and submerged in 0.1% sodium hypochlorite solution for 1 min, followed by rinsing with autoclaved seawater. The clean sori tissues were blotted dry using Kimtech absorbent papers, followed by overnight drying at 4°C. Spores were released the next day in 50-mL Falcon tubes by submerging the sori in 45 mL autoclaved seawater for 30 min. Spore density was checked using a Sedgwick Rafter chamber under inverted microscope at 10×. Spore solution was filtered through a two-stack cell strainer of 100 µm and 40 µm (Grenier Bio-one Ltd, UK) and cultures of were established in 1-L culture bottles in standard f/2 medium (media recipe at www.ccap.co.uk). Cultures were maintained as mixed gametophyte cultures (female: male ratio 3:1) at 10 °C under red light (20 μmol photons m^−2^ s^−1^, 12:12 light: dark photoperiod) for >1 year, where media was replenished monthly.

### Experimental design

Prior to the experiments, *A. esculenta* gametophytes were filtered through a 40 µm cell strainer (Greiner), weighed and chopped into small fragments with a sterile razor blade. For every treatment combination, approximately, 0.1 g of *A*. *esculenta* gametophytes (mixed cultures with a female: male ratio of 3:1) were cultured in 150 mL medium in pre-weighed autoclaved glass dishes with lids.

A full factorial experimental design was used with three factors: light (blue at 455 nm wavelength and red at 631 nm wavelength), media (PES and f/2) and iron (present and absent). Every treatment combination was replicated 5 times, resulting in a total of 40 experimental replicates. Blue and red light was supplied by LED lights (LED strip light, Waterproof 20M, 5050, UK) in two separate growth chambers maintained at 8.8 ± 0.002 SE °C, light intensity (measured in PAR with a PG200N Spectral PAR meter, UPRtek, Taiwan) at 26.5 ± 1.7 SE µmol photons m^−2 ^s^−1^ with a 16:8 day:night cycle. PES media (Provasoli et al. 1957) was prepared according to Starr and Zeikus (1993), and f/2 media according to methods available on www.ccap.co.uk. For both types of media, stock was prepared with and without the addition of iron (for iron-free PES, ammonium iron(II)-sulfate6-hydrate and iron(III)chloride hexahydrate were left out; for iron-free f/2, iron(III) chloride hexahydrate was left out of the trace metal mix). For each experimental replicate, the gametophytes were cultured in 150 mL autoclaved UV-filtered seawater (FSW) supplemented with PES or f/2 stocks (PES + iron, PES – iron, f/2 + iron, f/2 – iron) for two months under experimental conditions. During this time, media was partially replenished every 2 weeks. After two months, Fv/Fm readings were taken from each replicate using a FP100 FluorPen (PSI) with 15 min of dark adaptation, by directly putting the probe on the submerged cultures. Three technical replicate readings were taken with each dish to account for differences in density throughout the dishes. Following this, each culture was examined under an Axio Observer A.1 (Zeiss) inverted microscope at ×20 magnification and 5 pictures per replicate were taken using an Axiocam 705. ImageJ was used to count number of males and females, number of oogonia and to mark whether sporophytes were absent or present. Lastly, the media was discarded through a 40 µm cell strainer (Greiner), excess water was removed by sucking all water from the biomass through the bottom of the filter with a disposable Pasteur pipette. The wet weight of each culture was recorded, and 0.1 g of biomass was harvested for pigment analysis.

### Pigment extraction

Pigment extraction methods were adapted from Wright and Foggo (2021). Each aliquot of 0.1 g (wet weight) *A. esculenta* biomass was homogenised in 1 mL of 90% acetone using micro pestles and a microtube homogeniser in 1.5 mL safe-lock microcentrifuge tubes (Eppendorf, Fisher Scientific, UK). The aliquots were vortexed, sonicated for 30 s and incubated overnight at 8 °C in darkness. Thereafter, the aliquots were centrifuged at 13,200 ×*g* for 5 min and 500 µL of supernatant was transferred to 24-well microplates (Greiner Bio-One Ltd, UK). Absorbance spectra were immediately recorded between 400 and 800 nm (1 nm resolution) with a POLARstar Omega multimode plate-reader (BMG Labtech). A blank of 90% acetone was used for every 5 samples in each microplate and duplicate readings were taken for every plate. The time taken for absorbance reading was negligible (< 1 min per microplate). The raw wavelength data was convoluted to µg pigment g^−1^ fresh weight in R studio (R Core Team 2022) using the formulae and code from Wright and Foggo (2021) for chlorophyll *a* (Chl *a*), chlorophyll *c* (Chl *c*) and fucoxanthin contents. Chl *c* content was calculated as the sum of Chl *c*_1_ and Chl *c*_2_ and the antenna pigment to Chl *a* ratio was calculated as (Fucoxanthin + Chl *c*)/Chl *a* (Delebecq et al. 2013). Trace pigments ββ-carotene and zeaxanthin were grouped together as minor carotenoids as they exhibit identical absorption spectra (Wright and Fogo 2021).

### Statistical analysis

All statistical analysis was carried out using R studio (R Core Team 2022). All univariate data was analysed using generalized linear models (GLMs) fitted to test the effect of light, media-type and iron addition and their interaction on the response variables (weight, F_v_/F_m_, sex ratio, number of oogonia, number of healthy and malformed sporophytes and individual pigment content). Models were tested for main and interactive effects with analysis of deviance assessments (type III Wald chi-square test) using likelihood ratios in the *car* package (Fox and Weisberg 2019). If significant interactions were present, they were followed up by pairwise comparisons using estimated marginal means with a Tukey correction for multiple testing using the *emmeans* package (Russell 2024). Gametophyte weight and individual pigment content were fitted with an identity link function (Gaussian distributed response), and number of oogonia, sex ratio and sporophytes as well as F_v_/F_m_ were fitted using a logistic link function (binomially and Poisson distributed response variables). Sex ratio as a response variable was structured in a matrix containing male and female counts for each replicate. Residual plots were assessed visually to confirm the GLMs satisfied assumptions of homogeneity of variance.

Multivariate data on total pigment composition was analysed with a permutational multivariate analysis of variance using the *vegan* package (Oksanen et al. 2022). Analysis was based on Bray-Curtis distances between samples based on total pigment composition. A SIMPER analysis was done on the compositional pigment data to determine which pigments drove the changes in composition, using the *vegan* package (Oksanen et al. 2022). Any significant interactions were followed by pairwise comparisons of contrasts calculated using the package *pairwise.Adonis* (Martinez Arbizu 2022). Each pigment was also analysed as a univariate response by fitting GLMs with an identity link function, as per the method above. All data visualisation was done using the *ggplot2* package (Wickham 2016).

## Results

### Gametophyte growth

**Fig. 1:**
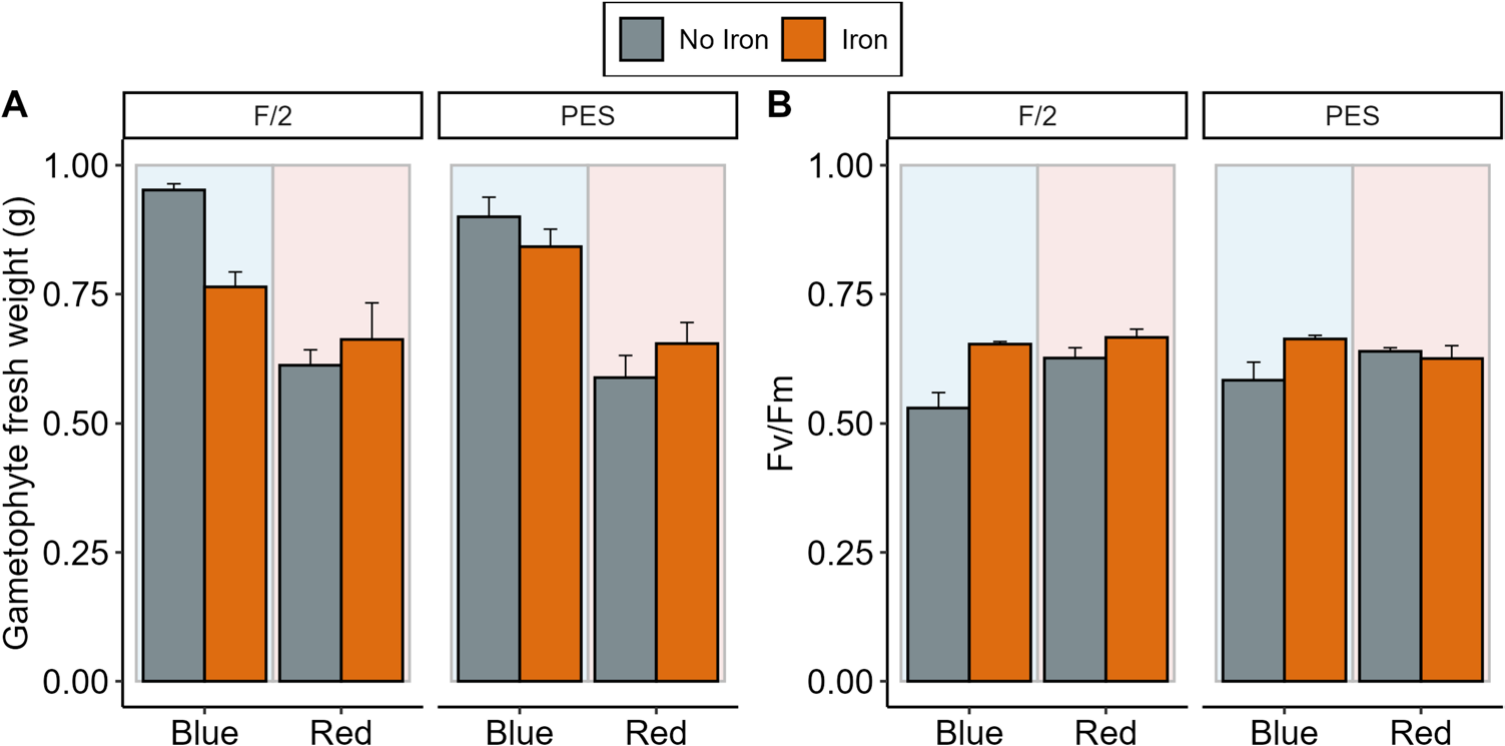
*Alaria esculenta* gametophyte A) weight and B) F_v_/F_m_ under different light (blue and red), media (f/2 and PES) and iron (present and absent) treatments. Error bars represent standard error (*n* = 5).

The final weight of *A*. *esculenta* gametophytes differed between light and iron treatment and their interaction (Fig. 1A, Table 1, χ^2^ = 8.68_1,32_, *p* = 0.003). The wet weight of gametophyte biomass increased nearly 10-fold during the 2-month culture period, where cultures started with 0.1 g of fresh weight and the final weights varied between ~0.60-0.95 g. The type of medium did not significantly affect the final weight of gametophyte biomass (Fig. 1A, Table 1, χ^2^ = 0.83_1,32_, *p* = 0.36). Gametophytes grown under red light had a lower biomass (Fig. 1A, Table 1, *p* < 0.001) compared to those grown under blue light. Iron had a significant effect on gametophyte weight only under blue light, where gametophyte cultures obtained a higher biomass when cultivated without iron (Fig. 1A, Table 1, *p* = 0.004).

**Table 1:** Type III Wald chi-square test of GLMs modelling on weight, F_v_/F_m_, sex ratios and ontogenic developments of *A. esculenta* gametophytes cultivated under different light (blue and red), media (f/2 and PES) and iron (present and absent) treatments. Significant results at α = 0.05 level are indicated in bold. A post-hoc comparison was performed for significant interactions using estimated marginal means with a Tukey correction for multiple testing.

*Variable*	*Factor*	*Χ*^*2*^	*df*	*p*	*posthoc*
Weight	Iron	10.832	1	**<0.001**	
	Media	0.829	1	0.363	
	Light	35.428	1	**<0.001**	
	Iron x Media	2.590	1	0.108	
	Iron x Light	8.680	1	**0.003**	*Blue: No iron > Iron* *Red: No iron = Iron* *Blue > Red*
	Media x Light	0.120	1	0.729	
	Iron x Media x Light	0.996	1	0.318	
Fv/Fm	Iron	0.156	1	0.692	
	Media	0.028	1	0.867	
	Light	0.095	1	0.758	
	Iron x Media	0.008	1	0.928	
	Iron x Light	0.033	1	0.856	
	Media x Light	0.008	1	0.931	
	Iron x Media x Light	0.001	1	0.980	
M:F	Iron	0.057	1	0.811	
	Media	0.867	1	0.352	
	Light	0.057	1	0.811	
	Iron x Media	0.350	1	0.554	
	Iron x Light	0.042	1	0.837	
	Media x Light	0.488	1	0.485	
	Iron x Media x Light	0.020	1	0.888	
Oogonia	Iron	4.388	1	**0.036**	*Iron > No iron*
	Media	0.016	1	0.900	
	Light	3.251	1	0.071	
	Iron x Media	0.001	1	0.976	
	Iron x Light	0.361	1	0.548	
	Media x Light	0.001	1	0.970	
	Iron x Media x Light	0.295	1	0.587	
Sporophyte	Iron	0	1	1	
	Media	0	1	1	
	Light	13.863	1	**>0.001**	*Blue > Red*
	Iron x Media	0	1	1	
	Iron x Light	0	1	1	
	Media x Light	0	1	1	
	Iron x Media x Light	0	1	1	
Malformed	Iron	3.855	1	**0.050**	*Iron > No iron*
	Media	8.456	1	**0.004**	*PES > f/2*
	Light	1.498	1	0.221	
	Iron x Media	0	1	0.999	
	Iron x Light	0.595	1	0.440	
	Media x Light	0	1	0.999	
	Iron x Media x Light	0	1	0.999	

F_v_/F_m_ was not significantly affected by either light (Fig. 1B, Table 1, χ^2^ = 0.09_1,32_, *p* = 0.76), medium type (Fig. 1B, Table 1, χ^2^ = 0.03_1,32_, *p* = 0.87) or iron treatments (Fig. 1B, Table 1, χ^2^ = 0.16_1,32_, *p* = 0.69). An average F_v_/F_m_ of 0.62 ± 0.02 SE indicated that the cultures were healthy and not photosynthetically stressed across all treatments.

### Pigments

**Fig. 2:**
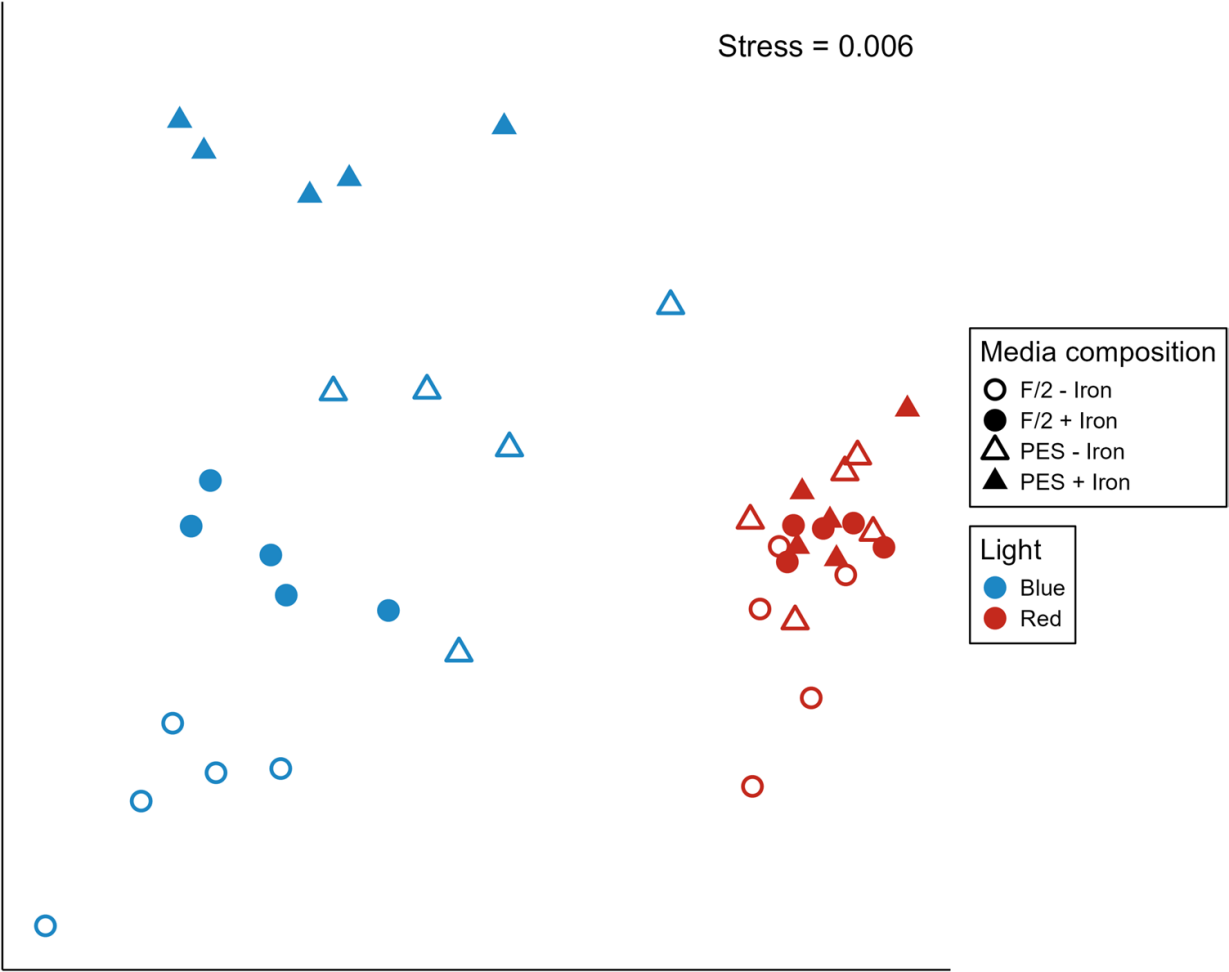
Non-metric multidimensional scaling (nMDS) ordination of pigment composition (based on Bray-Curtis distance) of *A. esculenta* gametophytes cultivated under different light (blue and red), media (f/2 and PES) and iron (present and absent) treatments (*n* = 5). Stress = 0.006.

The overall pigment composition was significantly affected by light (pseudo-F_1, 32_ = 365.45, *p* < 0.001) and type of medium (pseudo-F_1, 32_ = 16.98, *p* < 0.001), as well as the interaction between light, medium type, and presence of iron (Fig. 2, Table 2, pseudo-F_1, 32_ = 5.13, *p* = 0.03). Post hoc comparisons showed that under red light, there is no difference in pigment composition between medium and iron treatments (Fig. 2, Table 2, *p* = 0.18). However, in blue light, an interaction between medium and iron showed that when iron is absent, pigment composition significantly differed between f/2 and PES treatments (Fig. 2, Table 2, *p* = 0.01). In addition, among the two media investigated, the pigment composition was only significantly affected by iron addition in f/2 media (Fig. 2, Table 2, p = 0.01). SIMPER analysis indicated that the difference in pigment composition is mainly driven by the Chl *a* and fucoxanthin content.

**Table 2:** Permutational analysis of variance based on Bray–Curtis similarity measures for photosynthetic pigment composition of *A. esculenta* gametophytes among differing light (blue and red), media (f/2 and PES) and iron (present and absent) treatments.

*Variable*	*df*	*Pseudo-F*	*p*
Iron	1	365.44	**0.0001**
Media	1	16.98	**0.0009**
Light	1	2.15	0.1449
Iron x Media	1	9.83	**0.0039**
Iron x Light	1	2.28	0.1422
Media x Light	1	7.93	**0.0078**
Iron x Media x Light	1	5.13	**0.0299**
*Posthoc contrasts*			
RED Fe = Media			
BLUE Iron present: f/2 = PES | Iron absent: PES ≠ f/2 PES: iron present = iron absent | f/2: iron present ≠ iron absent			

**Fig. 3:**
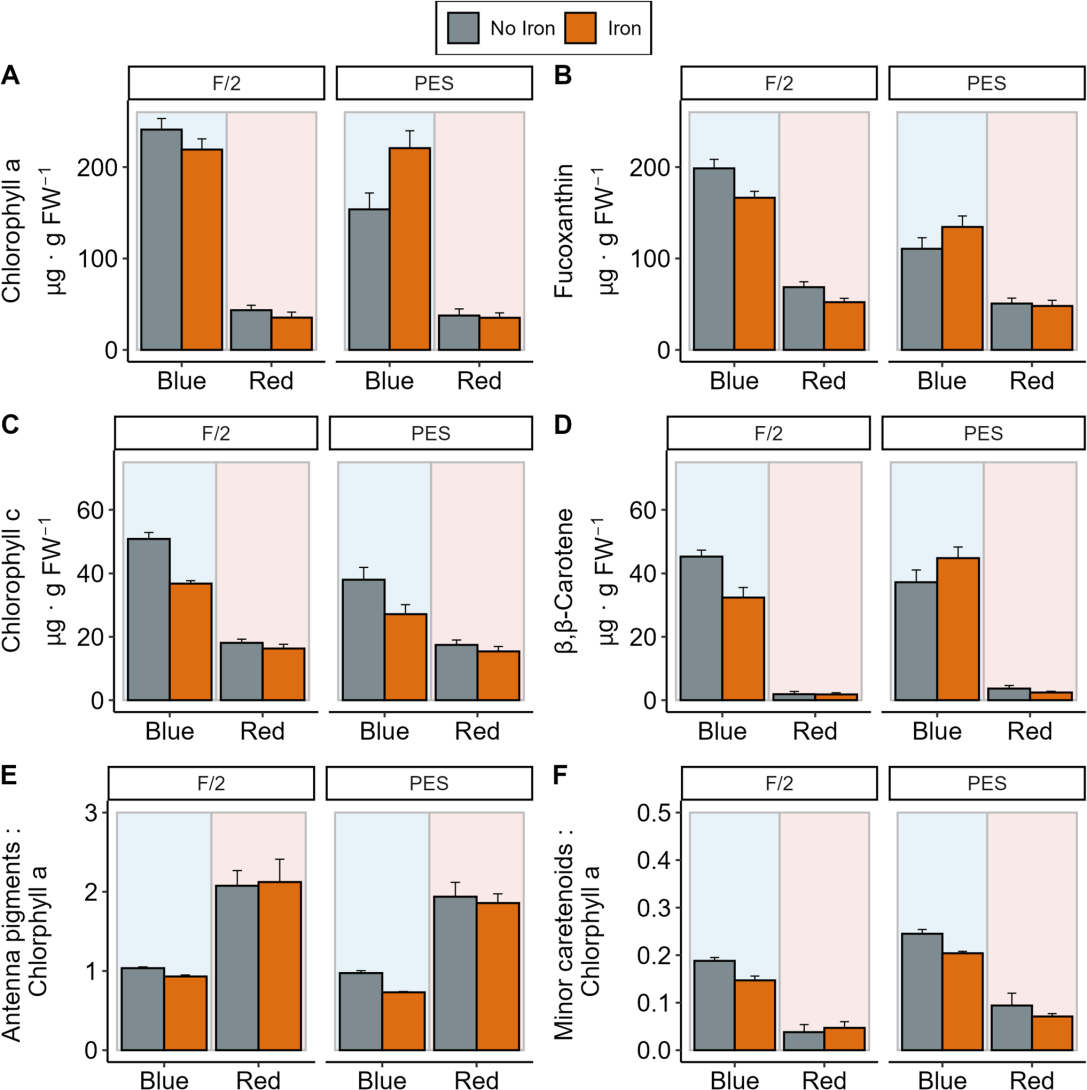
Individual pigment contents of *Alaria esculenta* gametophytes cultivated under different light (blue and red), media (f/2 and PES) and iron (present and absent) treatments: **A**) chlorophyll *a*; **B**) fucoxanthin; **C**) chlorophyll *c* and **D**) β,β-carotene. Error bars represent standard error (*n* = 5)

Analysis of variance on models of individual pigments also showed the strong effect of red and blue light on pigment contents of *A*. *esculenta* gametophytes, where the contents of Chl *a*, *c*, fucoxanthin and ββ-carotene were higher in blue light compared to red light (Fig. 3, Table S1). Post hoc comparisons of a three-way interaction between iron, light and media (Table S1, χ^2^_1, 32_ = 6.21, *p* = 0.013) showed that in blue light, adding iron only resulted in higher Chl *a* content in PES (Fig. 3A, Table S1, *p *<0.001). A two-way interaction for light and media as well as light and iron showed that Chl *c* and fucoxanthin content of gametophytes cultivated under blue light increased in f/2 medium compared to PES, and increased with iron addition (Fig. 3B-C, Table S1, *p *<0.001). A three-way interaction in β,β-carotene showed an opposite effect of medium and iron in gametophytes cultivated under blue light (Fig. 3D, Table S1, χ^2^_1, 32_ = 10.62, *p* = 0.001); the addition of iron in PES resulted in higher ββ-carotene content (p<0.001), while the addition of iron in f/2 decreased ββ-carotene content (Fig. 3D, Table S1, *p* = 0.02). The content of antenna pigments relative to Chl *a* was significantly higher by 117% in *A*. *esculenta* gametophytes cultivated under red light compared to blue light (Fig. 3E, Table S1, χ^2^_1, 32_ = 25.77, p < 0.001), while medium and iron had no significant effects (Fig. 3E, Table S1). On contrary, the content of minor carotenoids relative to Chl *a* was significantly higher in *A*. *esculenta* gametophytes cultivated under blue light as compared to red light (Fig. 3F, Table S1, χ^2^_1, 32_ = 66.21, *p* < 0.001), with higher contents in gametophytes cultivated in PES media as compared to f/2 (Fig. 3F, Table S1, χ^2^_1, 32_ = 9.27, *p* = 0.002) and without iron supplementation (Fig. 3F, Table S1, χ^2^_1, 32_ = 5.12, *p* = 0.023). The effect size of light was considerably larger than that of iron source or media (~200% increase from red to blue light, compared to ~20-% and ~45%, respectively).

### Gametophyte fertility and development

**Fig. 4:**
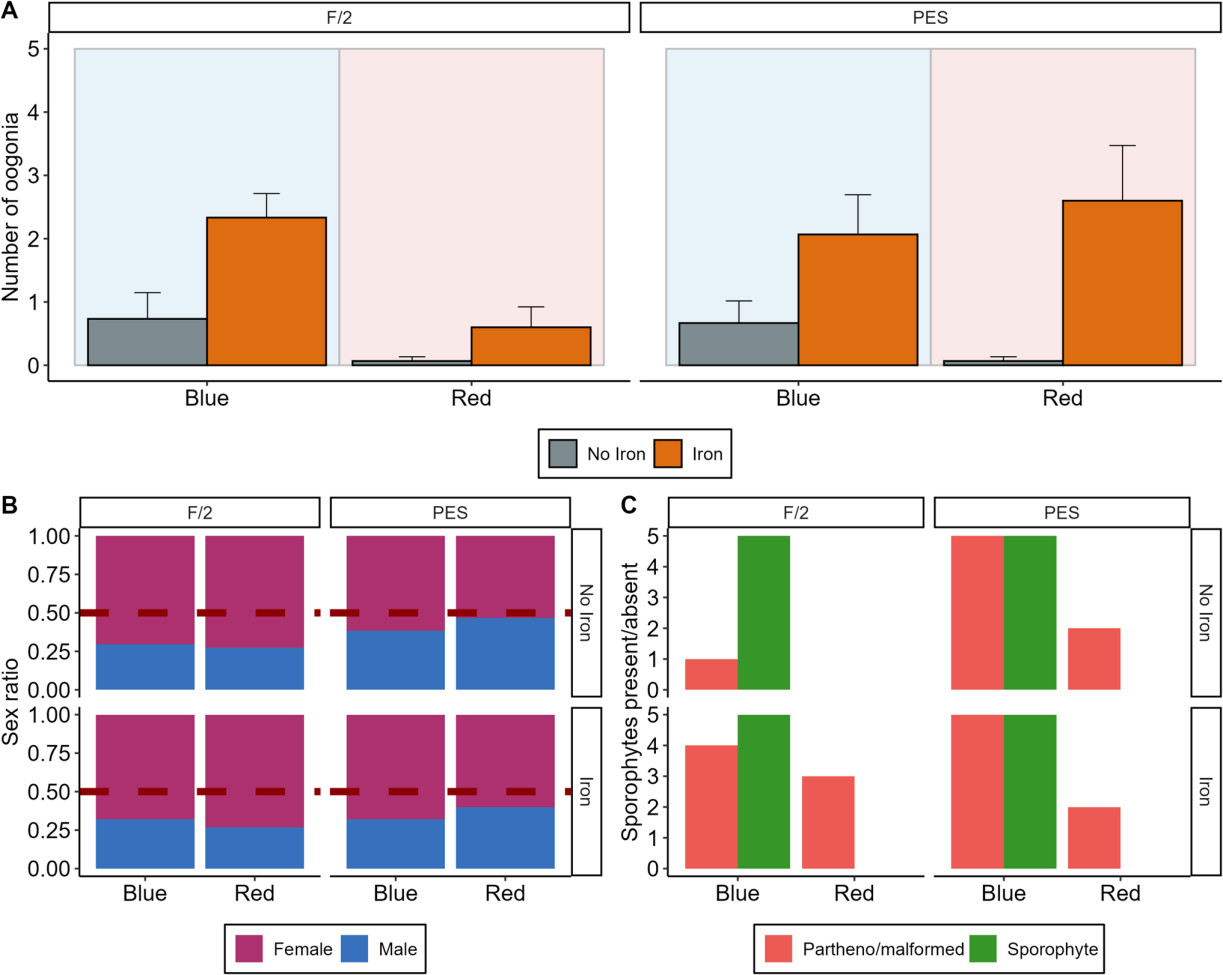
The effect of cultivating *Alaria esculenta* gametophytes under different light (blue and red), media (f/2 and PES) and iron (present and absent) treatments on **A**) number of oogonia; **B**) sex ratios and **C**) number of sporophytes (both normal and malformed). Error bars represent standard error (*n* = 5)

**Fig. 5:**
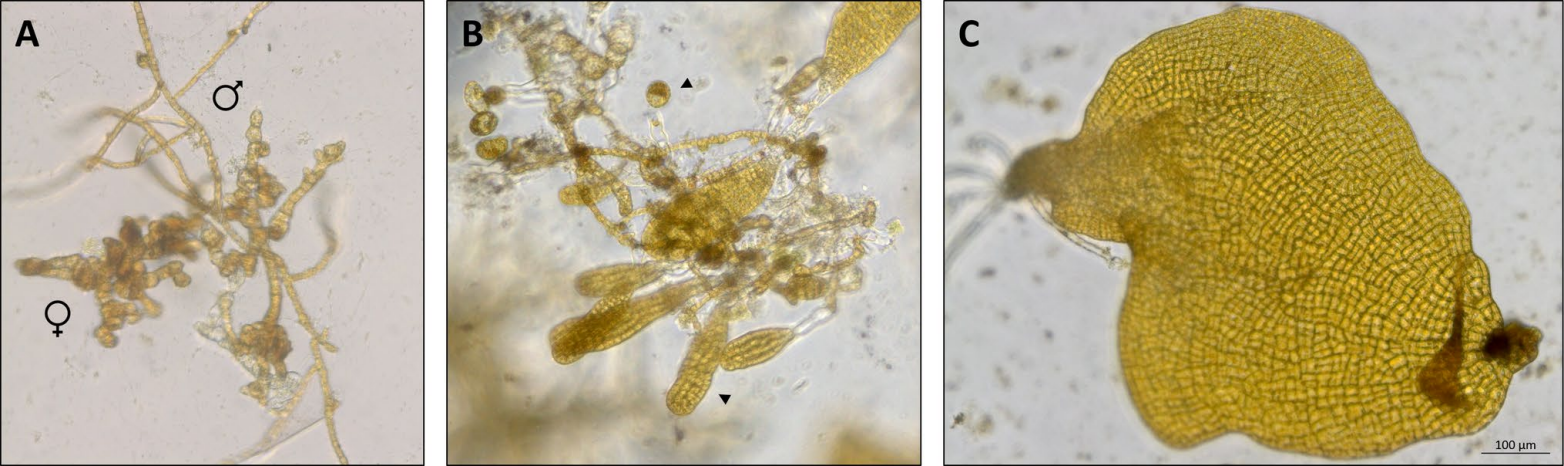
*Alaria esculenta* gametophyte developmental stages. **A**) male and female vegetative gametophyte as indicated by ♂ (male) and ♀ (female) symbols; **B**) a fertile female with oogonia and juvenile sporophytes attached as indicated by arrows; **C**) a malformed sporophyte as found in all cultures except those cultured under red light in iron-free f/2. The scalebar is 100 µm for all images (A-C)

The gametophyte cultures were assessed after two months for the presence of oogonia (Fig. 4A, 5B), sex ratio (Fig. 4B, 5A) and presence of any sporophytes (Fig. 4C, 5B). Sex ratio was not significantly changed by either light (Fig. 4B, Table 1, χ^2^_1, 32_ = 0.06, *p* = 0.81), medium (Fig. 4B, Table 1, χ^2^_1, 32_ = 0.87, *p* = 0.35) or iron treatment (Fig. 4B, Table 1, χ^2^_1, 32_ = 0.06, *p* = 0.81) from its initial 3:1 female: male ratio. However, number of oogonia produced by female gametophytes was significantly higher in treatments with added iron (Fig. 4A, Table 1, χ^2^_1, 32_ = 4.39, *p* = 0.04), but was not affected by either medium (Fig. 4A, Table 1, χ^2^_1, 32_ = 0.02, *p* = 0.90) or light (Fig. 4A, Table 1, χ^2^_1, 32_ = 3.25, *p* = 0.07). Light affected the development of healthy sporophytes where sporophytes developed in all replicates in blue light, while none developed in experimental replicates cultivated under red light (Fig. 4C, Table 1, χ^2^_1, 32_ = 13.86, *p*<0.001). However, malformed sporophytes (Fig. 5C) were present in both red and blue light treatments, except in replicates cultivated in f/2 without iron supplementation under red light (Fig. 4C). There were significantly more malformed sporophytes present in treatments with iron compared to no iron (Fig. 4C, Table 1, χ^2^_1, 32_ = 3.85, *p*=0.05), as well as more in treatments with PES as compared to f/2 (Fig. 4C, Table 1, χ^2^_1, 32_ = 8.46, *p*=0.004). Note that the χ^2^ values for malformed and healthy sporophytes are 0 for some treatments, which is due to some treatment combinations having 0 datapoints as not all treatments had either healthy or malformed sporophytes. Therefore, the statistical analysis should be interpreted with care.

## Discussion

In order to sustainably develop the North Atlantic seaweed industry and meet the rising demand for high quality seed stock, strategies should be developed to increase the efficiency at the nursery stage of kelp cultivation. Here we have tested the effect of light, nutrient media and iron supplementation on the growth, pigment composition and fertility of one-year delayed *A*. *esculenta* gametophytes. We found that blue light stimulated both growth and fertility in gametophytes. Withholding iron in blue light resulted in even higher growth and less malformed sporophytes but did not suppress the formation of healthy sporophytes. Pigment composition was also strongly influenced by light, with generally higher pigment content observed in gametophytes cultivated under blue light treatment and media and iron affecting compositional changes within blue light treatments only. Based on these results, we show a significant role of light source and iron supplementation in the cultivation cycle of *A. esculenta*.

### Growth

Increasing biomass during the bulking phase in the nursery may be achieved by supplying blue instead of red light, where final biomass was higher by 40% in the blue light treatment. This increase may have also been caused by the growth of juvenile sporophytes in the blue light treatments, which are generally larger than gametophytes. Blue light treatments achieved a dense mixed growth of both sporophytes and gametophytes, indicating that gametophytes also benefited from blue light supply. Additional variation in our growth measurements may have been caused by increased water mass in the higher density cultures, potentially increasing some of the difference found between the blue and red light treatments. However, it has been shown that blue light promotes the growth of both gametophytes and sporophytes (Cuijuan et al. 2005). The highest growth was achieved by withholding iron from the media, which may have been correlated to a lower number of malformed sporophytes in the iron-free treatment. The type of medium had no significant effect on the growth of gametophytes indicating that gametophyte growth is not limited by medium type but rather by light and availability of iron. This effect on growth has been found in *Saccharina japonica* cultivated in different growth media (Ratcliff et al. 2017), but f/2 yielded larger female gametophytes in *Ecklonia radiata* compared to PES (Praeger et al. 2022). Preference for culture media may thus be species-specific. F_v_/F_m_ did not differ between any treatments, indicating cultures were not photosynthetically stressed. In *Alaria crassifolia*, F_v_/F_m_ measurements were higher in juvenile sporophytes compared to gametophytes (Borlongan et al. 2019), but here there was no difference in F_v_/F_m_ between light sources, which may serve as a proxy for life stage. Effective F_v_/F_m_ is influenced not only by light source but also light strength, and the light levels used in this experiment (~30 µmol photons m^−2^ s^−1^) is within the range of light experienced by gametophytes and juvenile sporophytes in the field under adult canopy, ranging from 5-50 µmol photons m^−2^ s^−1^ (Smith et al. 2022). Higher light stress may show a more pronounced difference between gametophyte and sporophyte F_v_/F_m_, where stage-specific photo-inhibition can take place (Fain and Murray 1982; Altamirano et al. 2004).

### Pigment composition

The photosynthetic apparatus of kelps is highly adaptable, and the composition of pigments contained within kelp tissue can change according to depth, light intensity and nutrient composition (Colombo-Pallotta et al. 2006; Fernandes et al. 2016; Labbé et al. 2024; Singh et al. 2024). Here we show a strong effect of light wavelength on pigment composition and quantity. Higher overall pigment concentration in blue light indicated a higher photosynthetic efficiency of gametophyte cultures in blue light, which is often associated with ‘shade-adapted’ plants (Altamirano et al. 2003). As the blue light cultures had a much higher concentration of juvenile sporophytes, this may be used as a proxy for the difference in pigment composition between juvenile sporophytes and gametophytes. Juvenile sporophytes have been found to be more shade-adapted than gametophytes and adult sporophytes both, and more sensitive to high-light stress (Altamirano et al. 2004; Roleda et al. 2007; Veenhof et al. 2022). This has been hypothesized to be related to differences in morphology, where the denser tuft-like growth of gametophytes may help protect against high light stress through self-shading, whereas juvenile sporophytes have long thin blade offering less protection (Altamirano et al. 2004). In the context of cultivation, this means gametophytes can benefit from high light conditions, thus supplying higher and a broader spectrum of light may be beneficial to bulking gametophyte biomass.

When diving into the specific differences in pigment concentration, it was found that gametophytes cultivated under red light maintained a lower but stable pigment composition between experimental conditions. However, in blue light, juvenile sporophytes and gametophytes’ pigment composition changed with iron availability and type of media. Most pigments’ concentrations were higher in f/2 and adding iron into the media did not increase the pigment concentrations, while pigments were lower in PES and often would increase in quantity if iron was added (β,β-carotene, fucoxanthin, chlorophyll *a*). The better performance of f/2 over PES is also found in the cultivation of *Laminaria digitata* gametophytes, where f/2 results in higher growth of gametophytes only in blue light, but in red light f/2 and PES perform similarly (Ratcliff et al. 2017). Iron has been hypothesized to be an important part of the synthesis pathway of chlorophyll *a*, chlorophyll *b*, fucoxanthin and β,β-carotene in *Saccharina japonica* (Suzuki et al. 1994). There may be medium-specific differences in PES and f/2 where pigment development was supported by other trace metals than iron in f/2. For example, f/2 contains copper where PES does not (Starr and Zeikus 1993). While an excess of copper can disrupt photosynthesis and gametophyte development (Leal et al. 2016, 2018), copper is essential for normal functioning of the photosynthetic apparatus and chlorophyll synthesis (Droppa and Horváth 1990) and may have a role in pigment composition in kelp, based on our results. This media-specific effect of iron addition shows that using f/2 media may be preferred when withholding iron, as it does not affect the pigment composition as strongly as in PES.

The antenna pigment pool relative to chlorophyll *a* content was significantly higher in red-light gametophytes, while the xanthophyll cycle pool pigments relative to chlorophyll *a* were significantly lower in red light gametophytes. This is interesting as a larger pool of antenna pigments is typical of shade-adapted plants, as it increases photosynthetic efficiency (Erickson et al. 2015). A larger pool of xanthophyll cycle pigments is often associated with light-adapted plants, as these accessory pigments protects the photosynthetic apparatus from photodamage (Colombo-Pallotta et al. 2006; Delebecq et al. 2013). These two metrics then would indicate the red-light gametophytes more shade adapted than the blue light mix of juvenile sporophytes and gametophytes. This result contrasts with the overall higher pigment concentration of blue light juvenile sporophytes and gametophytes which indicates them to be more shade adapted. As both light treatments were grown in conditions representative of growth underneath adult canopy (Smith et al. 2022), the two contrasting responses may represent different strategies in adapting to low-light conditions. Juvenile sporophytes may increase their number of pigments in order to become more photosynthetically efficient in low-light environments, as has been found in adult sporophytes (Blain et al. 2020). Alternatively, gametophytes may increase their antenna size and decrease the carotenoid pool to increase photosynthetic capacity, another route to acclimate to low-light conditions (Franklin and Forster 1997; Wright and Foggo 2021).

### Fertility

The effects of red and blue light on kelp gametophyte fertility have both been well documented (Lüning and Dring 1975; Lüning 1981; Cuijuan et al. 2005) and our study confirmed that blue light stimulates the formation of sporophytes, while red light supresses sporophytes formation. However, in this study, malformed sporophytes were found in red light cultures, which suggested the production of parthenosporophytes via self-fertilization in female gametophytes. The formation of parthenosporophytes has been reported widely in kelps and can be characterized with irregular shapes, absence of polarity and no rhizoid formation (tom Dieck 1992; Müller et al. 2019) and is hypothesized to increase under stress or in marginalized populations (Oppliger et al. 2011; Camus et al. 2021). The increased number of malformed sporophytes with iron addition may simply be because iron stimulates egg formation and increased egg-counts may increase formation of parthenosporophytes. Alternatively, iron may actively stimulate parthenosporophyte formation. However, the mechanisms behind apomixis in kelps are complex and not yet fully resolved (Zhang et al. 2025). Interestingly, the only treatment combination where no malformed sporophytes were detected was under red light in f/2 medium without iron. Similarly, under blue light in f/2 without iron the ratio of healthy sporophytes to malformed ones was highest. Therefore, cultivating gametophytes in f/2 without iron may be beneficial not only for optimum biomass and pigment composition, but also for a low number of malformed sporophytes.

Withholding iron was not successful in repressing fertility in *A. esculenta* gametophyte cultures. This was contrary to what has previously been found on the essential role of iron in gametogenesis of a range of kelp species, where even in blue light no sporophytes are formed when iron is withheld (Lewis et al. 2013; Iwai et al. 2015; Chen et al. 2023). This means that either the function of iron in gametogenesis is species-specific, or gametophytes have some capacity for iron retention. As withholding of iron resulted in no egg formation in a closely related species of kelp, *A. marginata* (Lewis et al. 2013)*,* the latter seems more likely. Cultures that were used in this experiment had been cultured prior to the experiment in media with iron under red light. Iron could have been retained in gametophyte cells and used to facilitate egg production especially under blue light conditions. It has been suggested that copper can be retained in cell walls of gametophytes after copper has been taken out of the media (Leal et al. 2018), showing the capacity of gametophytes to retain trace metals within cellular tissues. Additionally, there could have been trace levels of iron present in the filtered autoclaved seawater itself, since the seawater used for this study was not treated to make it fully ‘iron-free’. Repeating this experiment with cultures that have been maintained without iron from the spore release stage could confirm this. There is also the possibility that gametophytes that have been in culture for 1+ year may react differently to light and nutrient cues than non-delayed gametophytes (Carney and Edwards 2010; Ebbing et al. 2025). As many nurseries work specifically with delayed gametophytes, the long-term effects of iron supplementation are especially important to understand.

## Conclusion

Here we demonstrate the effects of light source on the growth, pigment composition and fertility of *A. esculenta* gametophytes. While withholding iron did not fully supress sporophyte formation, it did cause less malformed sporophytes and higher biomass, especially in blue light. We therefore recommend cultivating gametophytes for bulk culture in f/2 medium without iron. In addition, pigment composition may change with light source and media, but higher pigment concentrations in blue light in f/2 without iron can indicate higher photosynthetic efficiency. This combination of growing conditions may prove to increase yields in the nursery, which in turn will help commercial kelp nurseries meet the rising demand for high quality and readily available seedstock.

## Supplementary Information

Below is the link to the electronic supplementary material.

Supplementary file1 (DOCX 21 KB)

## Data Availability

Availability of data and material – Data will be made available upon request

## Code availability

Code will be made available upon requests
