# Supplementary material for: Optimizing nursery conditions of the commercial kelp Alaria esculenta
Source: J Appl Phycol. 2025 Sep 27;37(5):3031–42. doi: 10.1007/s10811-025-03665-z (PMC12672681; doi:10.1007/s10811-025-03665-z)
Supplement: Supplementary file 1 — Supplementary file1 (DOCX 21 KB) [file 10811_2025_3665_MOESM1_ESM.docx]

**Supplementary materials**

**Optimizing nursery conditions of the commercial kelp *Alaria esculenta***

Reina J. Veenhof ^1^, Rob Grisenthwaite ^1, 2^, Alison Mair ^1^, Elaine Mitchell ^1^ Michele S. Stanley ^1^, Puja Kumari ^1^

^1^ Scottish Association for Marine Science, Oban, Argyll PA37 1QA, UK

^2^ SAMS Enterprise, Oban, Argyll PA37 1QA, UK

***** Corresponding author: Reina J. Veenhof ([reina.veenhof@uhi.ac.uk](mailto:reina.veenhof@uhi.ac.uk))

Table S1. Type III Wald chi-square test of GLMs modelling individual pigment quantity (µg · g FW^-1^) as well as antenna and minor caretenoid pigments relative to chlorophyll a concentration of *A. esculenta* gametophytes under differing light (blue and red), media (f/2 and PES) and iron (present and absent) treatments. Significant results at the α = 0.05 level are indicated in bold. If interactions are present, posthoc comparison was performed using estimated marginal means with a Tukey correction for multiple-testing.

| *Variable* | *Factor* | *F* | *df* | *p* | *posthoc* |
| --- | --- | --- | --- | --- | --- |
| Chlorophyll a | Light | 140.463 | 1 | **<0.001** |  |
|  | Media | 27.383 | 1 | **<0.001** |  |
|  | Iron | 1.712 | 1 | 0.191 |  |
|  | Light x Media | 11.911 | 1 | **0.001** |  |
|  | Light x Iron | 0.336 |  | 0.562 |  |
|  | Media x Iron | 14.181 | 1 | **<0.001** |  |
|  | Light x Media x Iron | 6.212 | 1 | **0.013** |  |
|  | Blue > Red  RED:  Media = Fe  BLUE:  Iron: F/2 > PES \| No iron: F/2 = PES  PES: Iron > No Iron \| F/2: Iron = No Iron | | | | |
| Chlorophyll c | Media | 17.691 | 1 | **<0.001** |  |
|  | Light | 115.819 | 1 | **<0.001** |  |
|  | Iron | 21.233 | 1 | **<0.001** | Blue > Red |
|  | Media x Light | 7.971 | 1 | **0.005** | Blue: No Iron > Iron  Red: No Iron = Iron |
|  | Media x Iron | 0.532 | 1 | 0.466 |  |
|  | Light x Iron | 8.083 | 1 | **0.004** | Blue: F/2 > PES  Red: F/2 = PES |
|  | Media x Light x Iron | 0.311 | 1 | 0.577 |  |
| Fucoxanthin | Media | 54.96 | 1 | **<0.001** |  |
|  | Light | 119.895 | 1 | **<0.001** |  |
|  | Iron | 7.336 | 1 | **0.007** |  |
|  | Media x Light | 17.421 | 1 | **<0.001** | Blue: F/2 > PES  Red: F/2 = PES |
|  | Media x Iron | 11.156 | 1 | **0.001** | F/2: No Iron > Iron  PES: No Iron = Iron |
|  | Light x Iron | 0.875 | 1 | 0.350 |  |
|  | Media x Light x Iron | 3.169 | 1 | 0.075 |  |
| β,β-Carotene | Media | 5.871 | 1 | **0.015** |  |
|  | Light | 172.027 | 1 | **<0.001** |  |
|  | Iron | 15.085 | 1 | **<0.001** |  |
|  | Media x Light | 4.385 | 1 | **0.036** |  |
|  | Media x Iron | 18.995 | 1 | **<0.001** |  |
|  | Light x Iron | 7.461 | 1 | **0.006** |  |
|  | Media x Light x Iron | 10.62 | 1 | **0.001** |  |
|  | Blue > Red  RED:  Media = Fe  BLUE:  Iron: PES > F/2 \| No iron: PES < F/2  PES: Iron > No Iron \| F/2: Iron < No Iron | | | | |
| Antenna pigments : Chlorophyll a | Media | 0.0917 | 1 | 0.762 |  |
|  | Light | 25.7743 | 1 | **<0.001** | Red > Blue |
|  | Iron | 0.2623 | 1 | 0.6085 |  |
|  | Media x Light | 0.069 | 1 | 0.7928 |  |
|  | Media x Iron | 0.2276 | 1 | 0.6333 |  |
|  | Light x Iron | 0.2769 | 1 | 0.5987 |  |
|  | Media x Light x Iron | 0.0008 | 1 | 0.9781 |  |
| Minor caretenoids : Chlorphyll a | Media | 9.272 | 1 | **0.002** | PES > F/2 |
|  | Light | 66.208 | 1 | **<0.001** | Blue > Red |
|  | Iron | 5.122 | 1 | **0.024** | No iron > Iron |
|  | Media x Light | 0.001 | 1 | 0.974 |  |
|  | Media x Iron | 0.001 | 1 | 0.975 |  |
|  | Light x Iron | 3.727 | 1 | 0.054 |  |
|  | Media x Light x Iron | 0.766 | 1 | 0.381 |  |
